# Publisher Correction: High-throughput characterization of HLA-E-presented CD94/NKG2x ligands reveals peptides which modulate NK cell activation

**DOI:** 10.1038/s41467-023-40946-y

**Published:** 2023-08-29

**Authors:** Brooke D. Huisman, Ning Guan, Timo Rückert, Lee Garner, Nishant K. Singh, Andrew J. McMichael, Geraldine M. Gillespie, Chiara Romagnani, Michael E. Birnbaum

**Affiliations:** 1 Koch Institute for Integrative Cancer Research, Cambridge, MA USA; 2 Department of Biological Engineering, MIT, Cambridge, MA USA; 3 Innate Immunity, Deutsches Rheuma-Forschungszentrum Berlin (DRFZ), ein Leibniz Institut, Berlin, Germany; 4 Centre for Immuno-Oncology, Old Road Campus Research Building, Nuffield Department of Medicine, University of Oxford, Oxford, UK; 5 Ragon Institute of MGH, MIT, and Harvard, Cambridge, MA USA; 6 Charité - Universitätsmedizin Berlin, Berlin, Germany

**Keywords:** MHC class I, Innate immunity, Peptides

Correction to: *Nature Communications* 10.1038/s41467-023-40220-1, published online 9 August 2023

The original version of this Article contained an error in Fig. 4, in which several datapoints did not display. This has been corrected in both the PDF and HTML versions of the Article.

