# Supplementary material for: High-throughput characterization of HLA-E-presented CD94/NKG2x ligands reveals peptides which modulate NK cell activation
Source: Nat Commun. 2023 Aug 9;14:4809. doi: 10.1038/s41467-023-40220-1 (PMC10412585; doi:10.1038/s41467-023-40220-1)
Supplement: Supplementary file 1 — Supplemental Information [file 41467_2023_40220_MOESM1_ESM.pdf]

Supplemental Figures

High-throughput characterization of HLA-E-presented CD94/NKG2x ligands reveals peptides which modulate NK cell activation

Brooke D. Huisman, Ning Guan, Timo Rückert, Lee Garner, Nishant K. Singh, Andrew J. McMichael, Geraldine M. Gillespie, Chiara Romagnani, Michael E. Birnbaum

14 Supplemental Figures
1 Supplemental Table

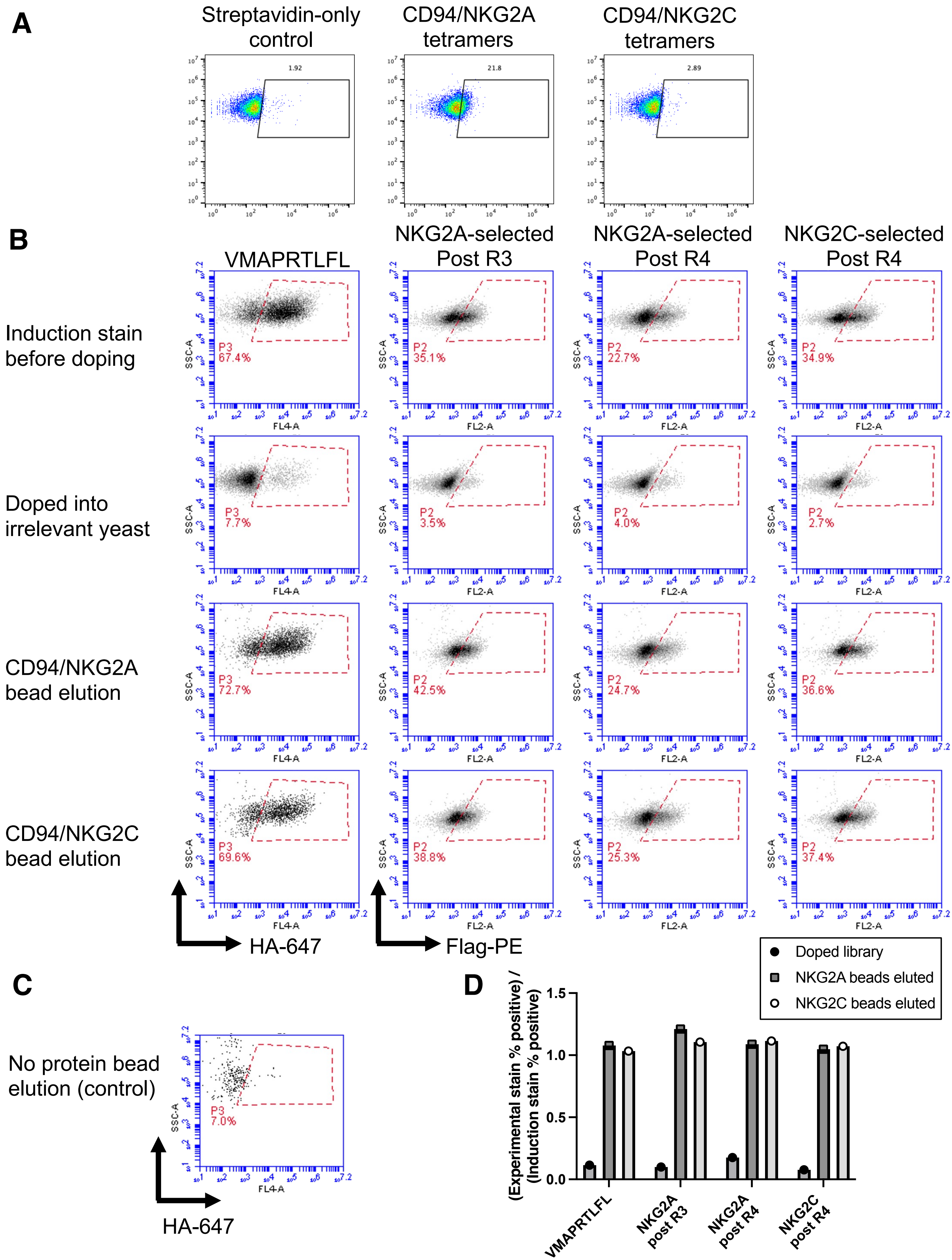

Supplemental Figure 1. Validation of HLA-E construct. *Continued on next page.*

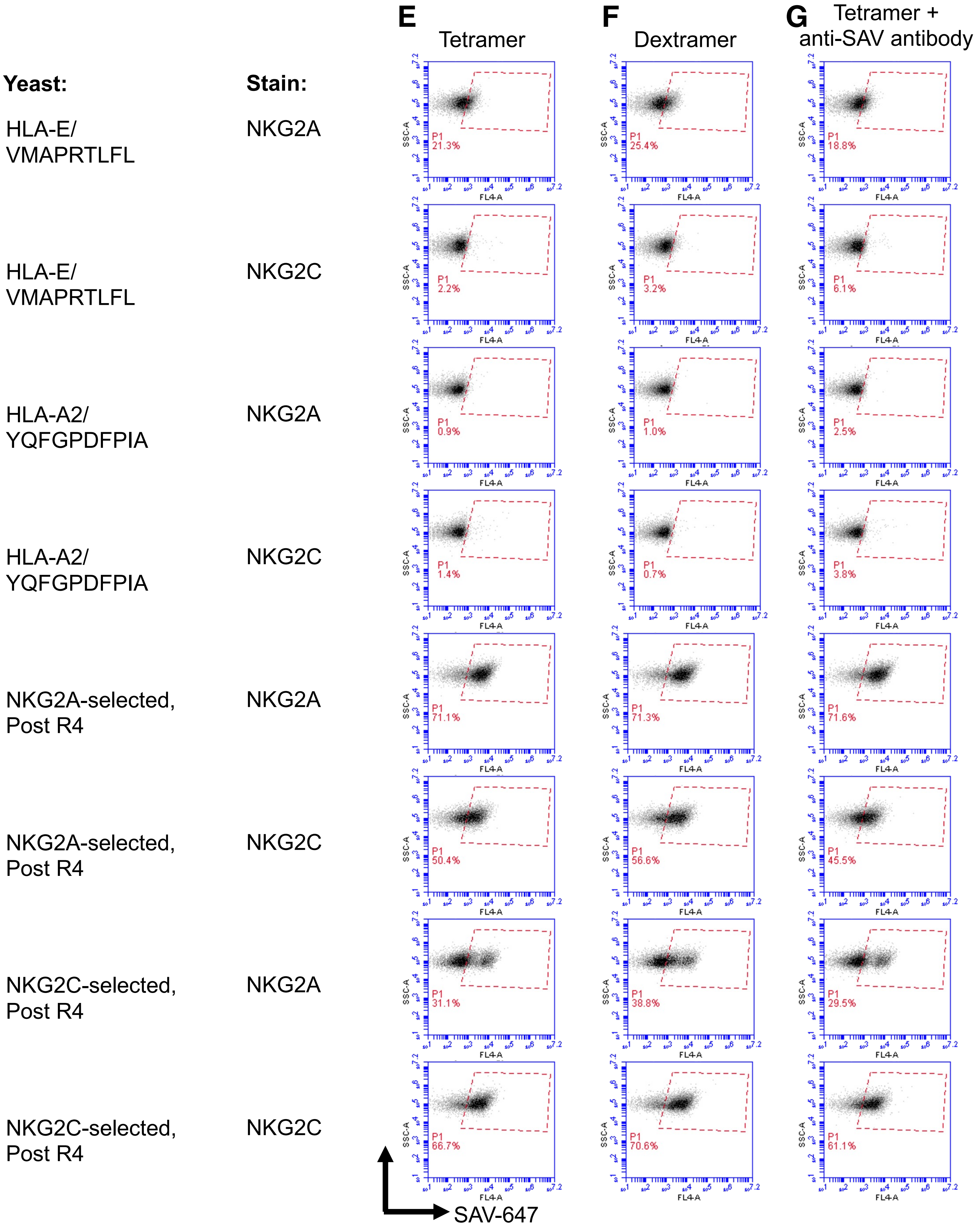

Supplemental Figure 1. Validation of HLA-E construct. *Legend on next page.*

Supplemental Figure 1 *(continued from previous page).* **Validation of HLA-E construct. a)** Validation staining of HLA-E with VMAPRTLFL peptide with CD94/NKG2A (same as **Figure 1**) and CD94/NKG2C tetramers, made with Streptavidin-647, with Streptavidin-647-only control. **b-d**) Column enrichment assay. **b)** VMAPRTLFL/HLA-E or selected HLA-E libraries were stained, doped into irrelevant unstained yeast, and enriched with CD94/NKG2A or CD94/NKG2C beads. **c)** Elution of VMAPRTLFL/HLA-E, enriched using beads without CD94/NKG2x, as a control. **d)** Efficiency of enrichment, as the post-enrichment percentage epitope tag-positive yeast divided by the pre-enrichment induction stain percentage epitope tag-positive yeast. **e-g)** Side-by-side staining of HLA-E/VMAPRTLFL, HLA-A2/YQFGPDFPIA (negative control), or post-round 4 selected yeast with CD94/NKG2x tetramer **(e),** dextramer **(f),** or tetramer with anti-streptavidin antibody **(g).** Source data are provided as a Source Data file.

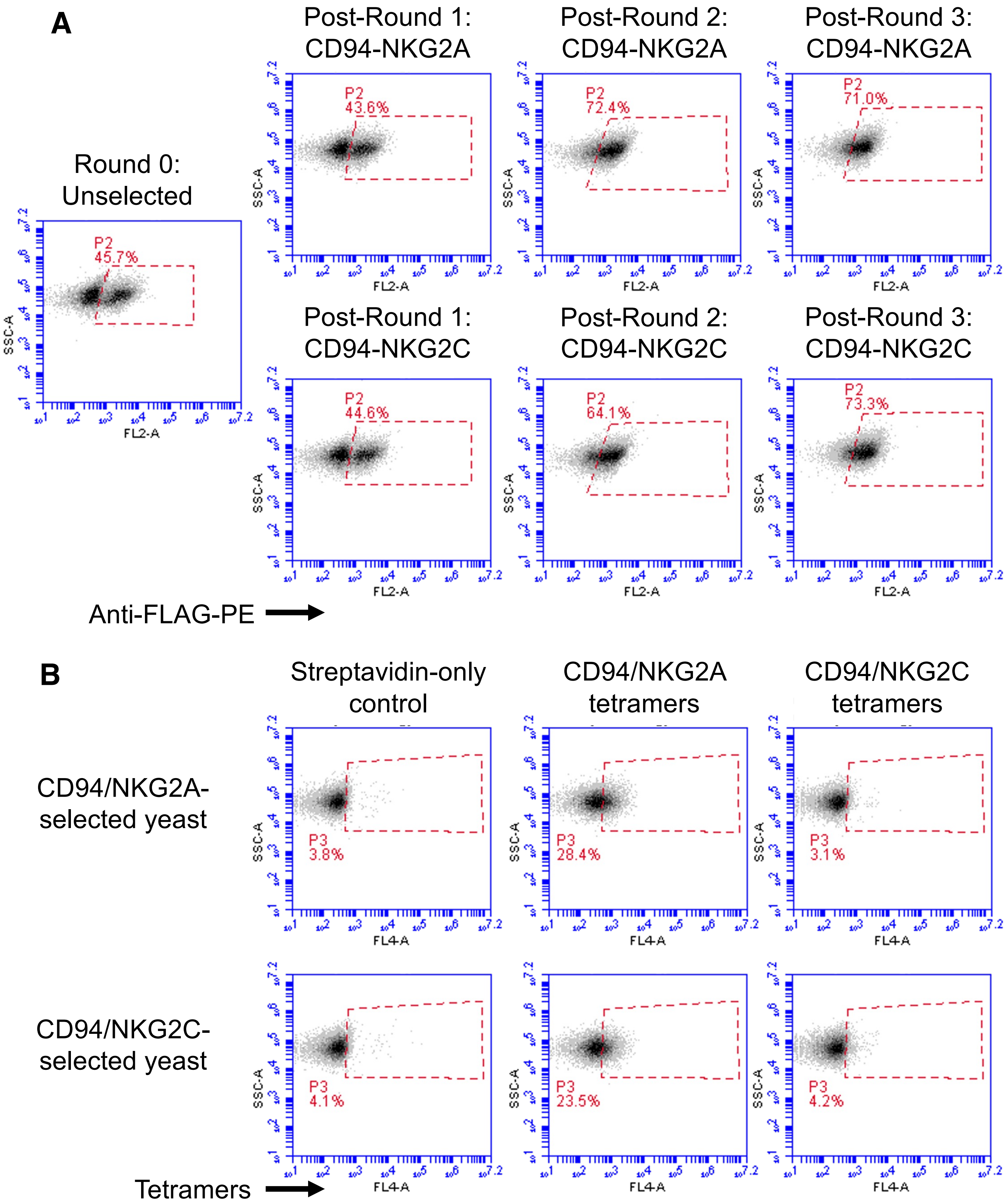

Supplemental Figure 2. Library dynamics over rounds of selection with CD94/NKG2A or CD94/NKG2C. a) Prior to selections, a sampling of yeast were assessed for FLAG epitope tag expression, which increased over rounds of selection (gate drawn daily on unstained yeast). **b)** Staining of yeast during Round 4 with CD94/NKG2A or CD94/NKG2C tetramers made with Streptavidin-647, on libraries previously selected with CD94/NKG2A or CD94/NKG2C in Rounds 1-3, including negative Streptavidin-647-only control.

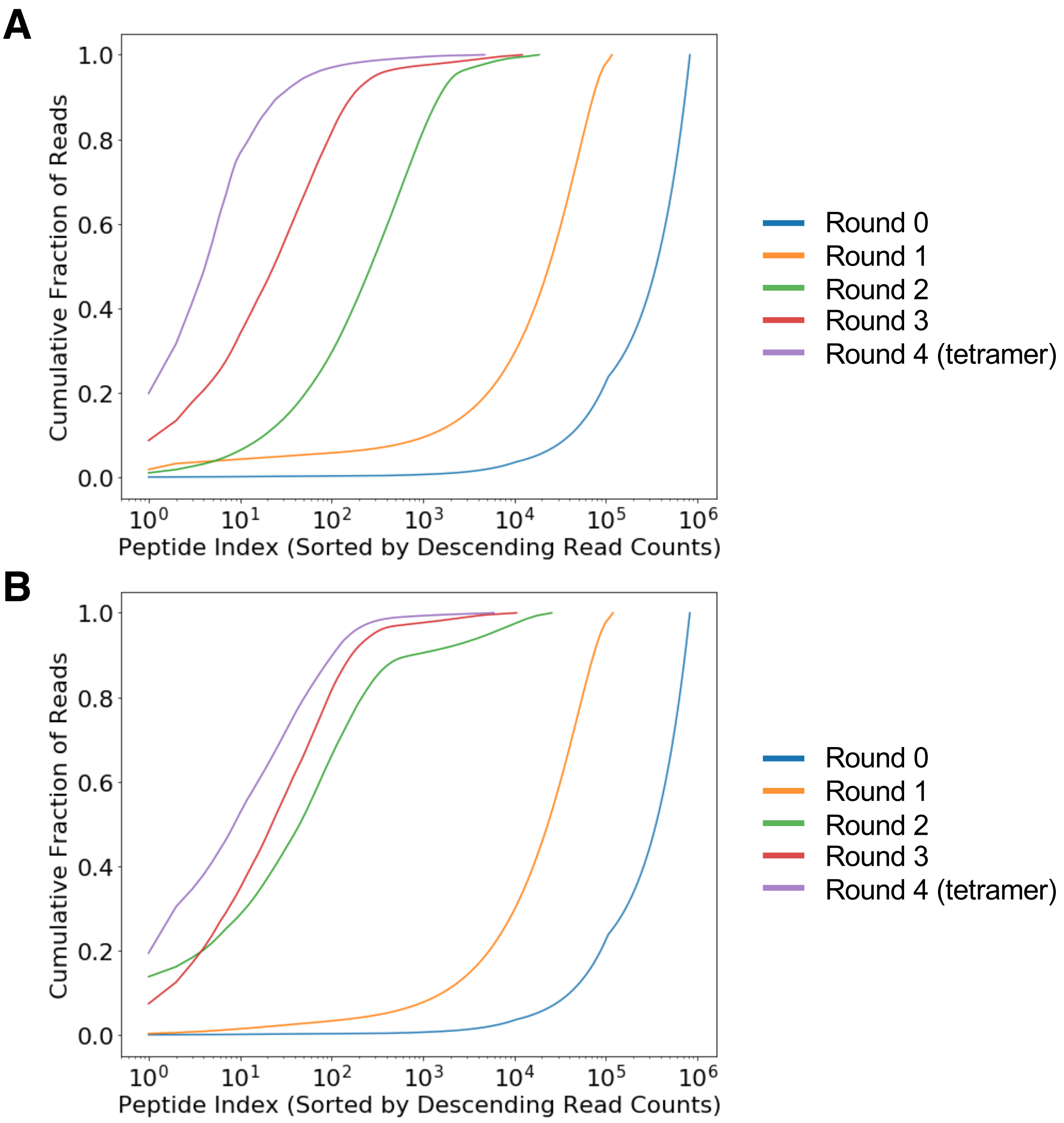

Supplemental Figure 3. Peptide frequency in deep sequencing data. Cumulative fraction of read counts for peptides selected with **a)** CD94/NKG2A or **b)** CD94/NKG2C. Source data are provided as a Source Data file.

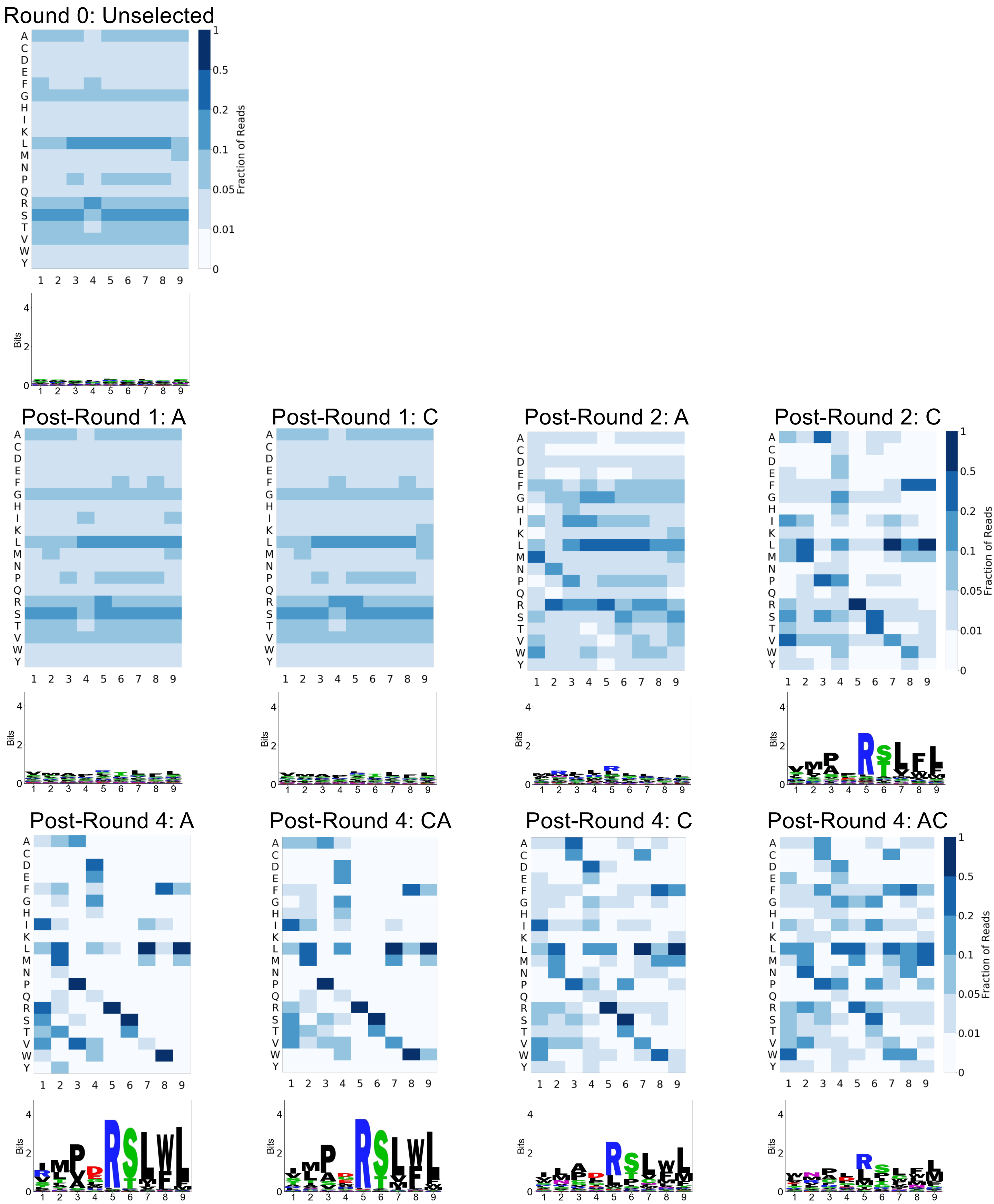

Supplemental Figure 4. Heatmaps and sequence logos for other rounds of selection. A: Selected with CD94/NKG2A; C: Selected with CD94/NKG2C; AC: yeast previously selected with CD94/NKG2A, cross-selected with CD94/NKG2C in this round; CA: yeast previously selected with CD94/NKG2C, cross-selected with CD94/NKG2A in this round. Source data are provided as a Source Data file.

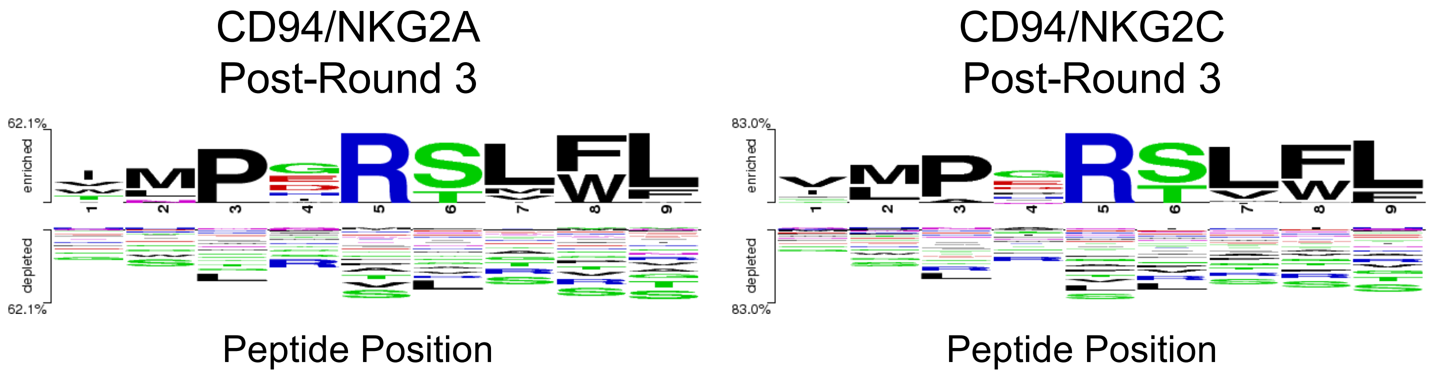

Supplemental Figure 5. Statistical analysis using Two Sample Logo. Statistically significant residues are shown, from CD94/NKG2A- or CD94/NKG2C-selected, post-round 3 libraries, compared to the unselected library. Significance was calculated by Two Sample Logo using the two-sided binomial test with Bonferroni correction and a cutoff of $p < 0.05$.

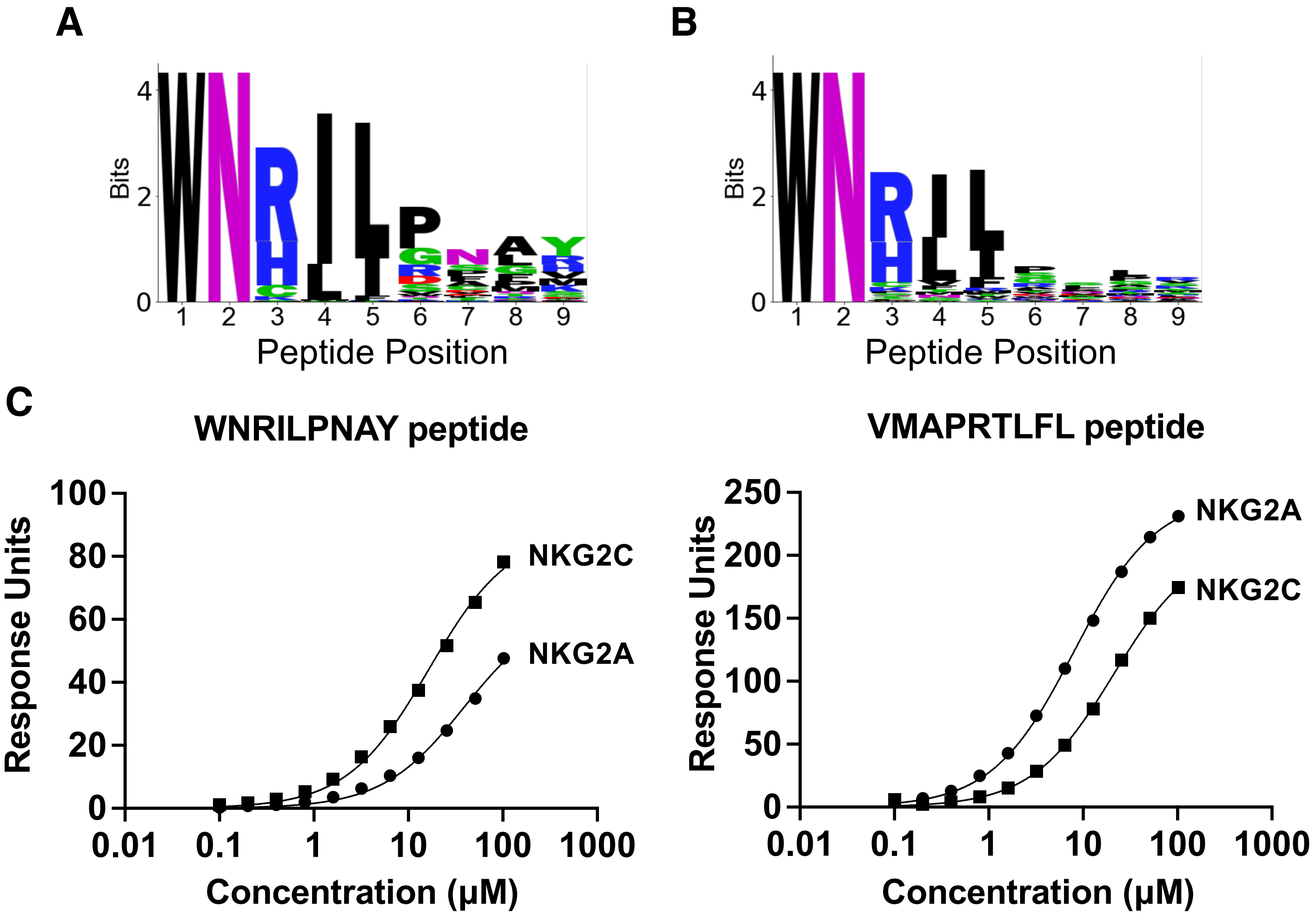

Supplemental Figure 6. Subdominant motif of peptides with P1 Trp and P2 Asn (WN peptides). Sequence motifs of peptides that have P1 Trp and P2 Asn, **a)** weighted by frequency or **b)** not weighted by frequency in Round 3 of selections with CD94/NKG2A. **c)** Surface plasmon resonance data for HLA-E single chain trimers containing WN 9mer peptide (left) or VMAPRTLFL control peptide (right), binding to CD94/NKG2A or CD94/NKG2C. Source data are provided as a Source Data file.

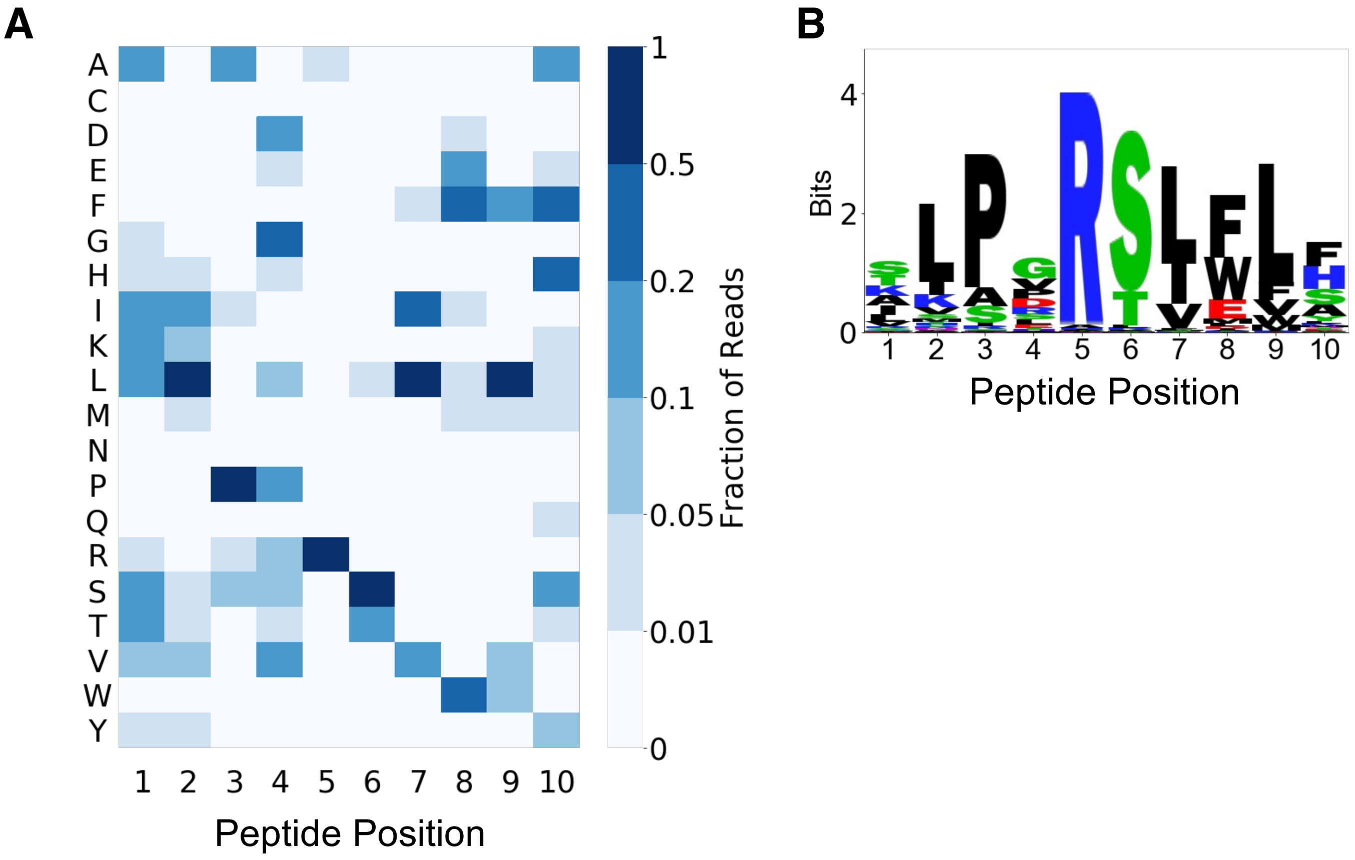

Supplemental Figure 7. 10mer peptide data a) Heatmap and **b)** sequence logo representation of peptide positional amino acid preferences in 10mer peptides after three rounds of selection with CD94/NKG2C, with peptides weighted by read count. Source data are provided as a Source Data file , and peptide sequences are provided in Supplemental Data 1.

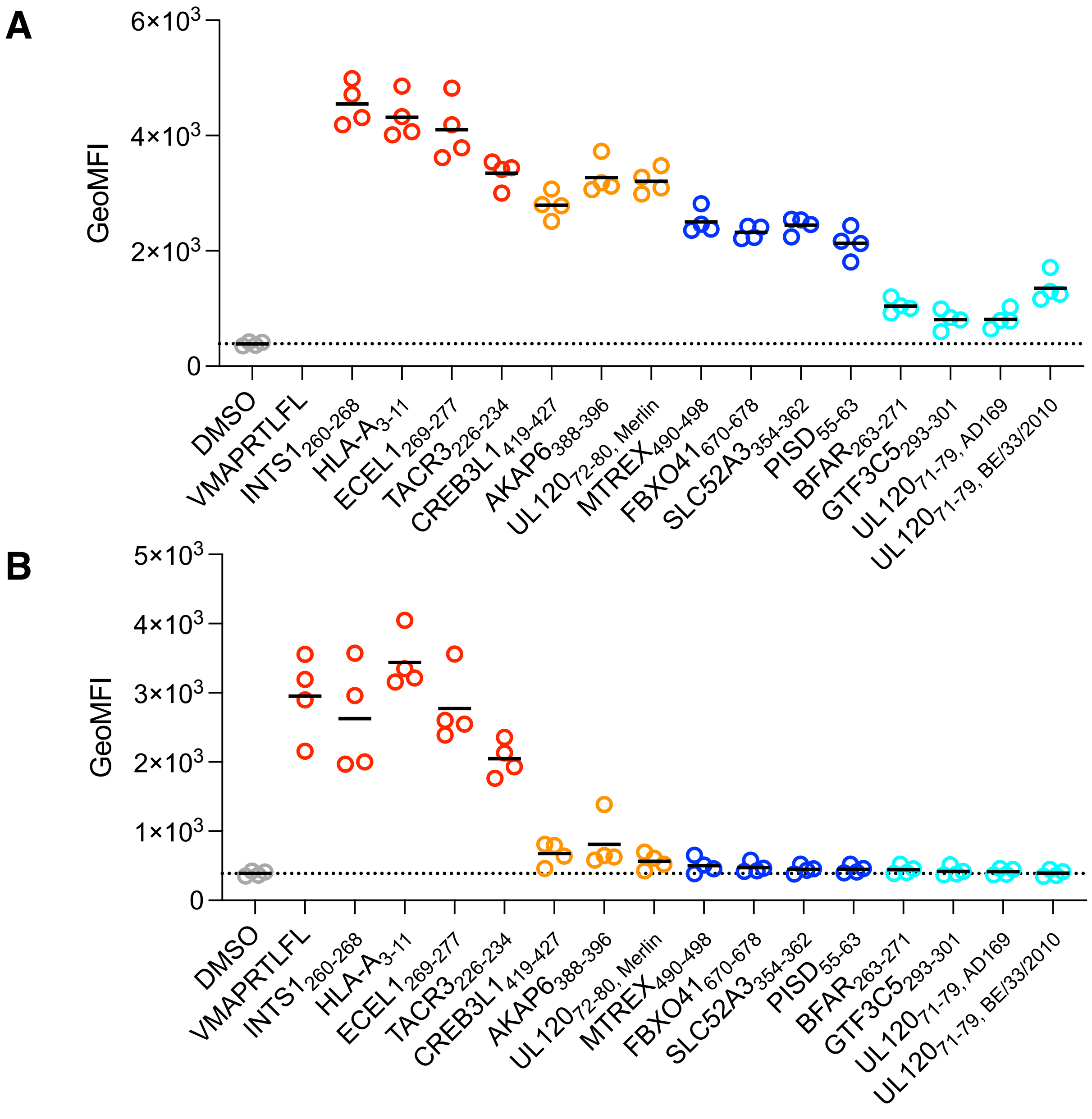

Supplemental Figure 8. Peptide stabilization of HLA-E surface expression at additional concentrations. Assessment of peptide-HLA-E binding via HLA-E surface stabilization assay with RMA-S/HLA-E cells incubated with **A)** 300 µM peptide (150 µM for $BFAR_{263\text{-}271}$ due to lower peptide solubility) and **B)** 3 µM peptide. Measurements from n=4 replicate experiments are shown, with solid black lines indicating mean values. Peptides with similar stabilization effects are plotted in the same color; in order from most to least stabilizing, the colors are: red, orange, blue, and cyan, with DMSO control in grey. Source data are provided as a Source Data file.

A

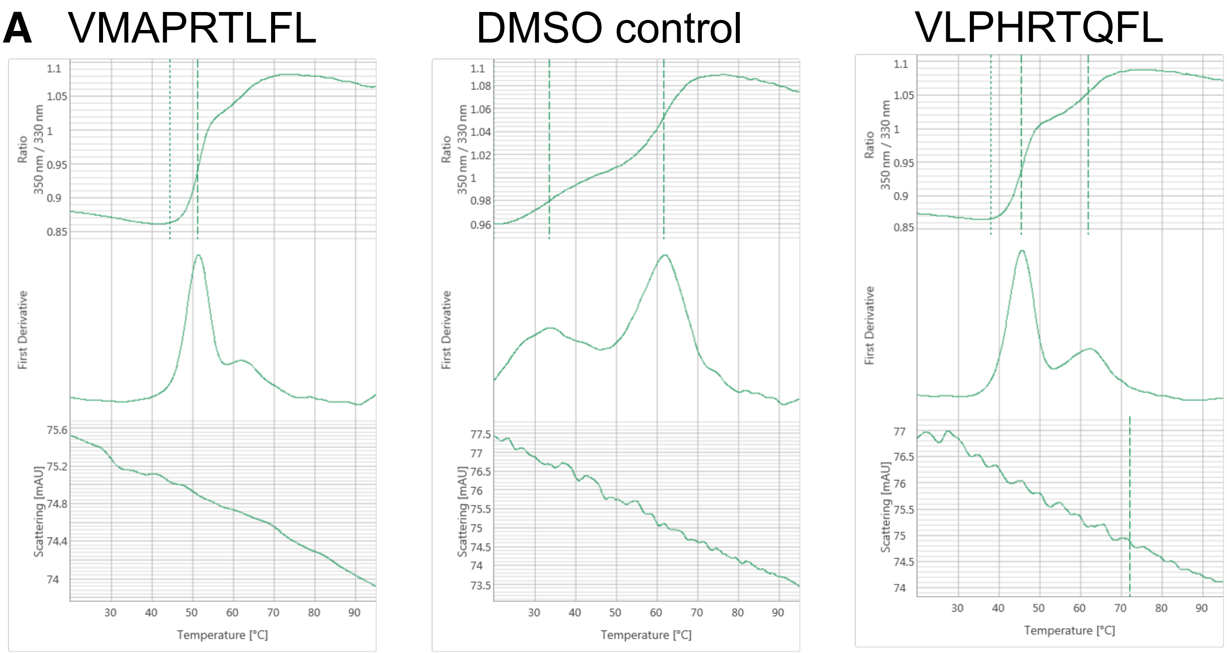

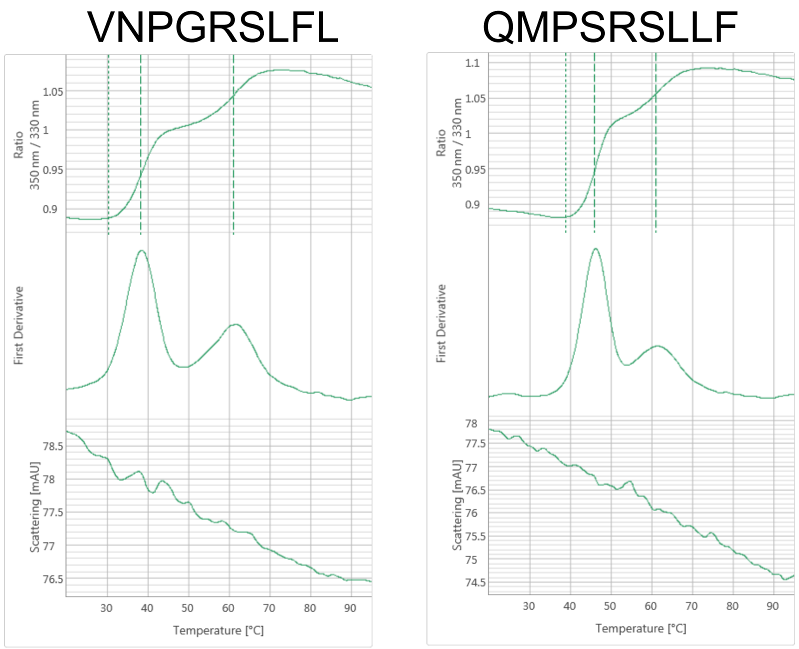

B

Peptide (Name)	T_m (°C)
VMAPRTLFL (control)	51.5
QMPSRSLLF (CREB3L1$_{419\text{-}427}$)	46.2
VLPHRTQFL (UL120$_{72\text{-}80,\ Merlin}$)	45.7
VNPGRSLFL (BFAR$_{263\text{-}271}$)	38.5
DMSO control	33.8

Supplemental Figure 9. Differential scanning fluorimetry data generated via Prometheus NanoTemper for predicted peptides with HLA-E. a) Raw 350/330 nm ratios and first derivative for samples. The second inflection point is consistent with β2M unfolding. **b)** Summary of T_m values for the first inflection point called by Prometheus software for these samples.

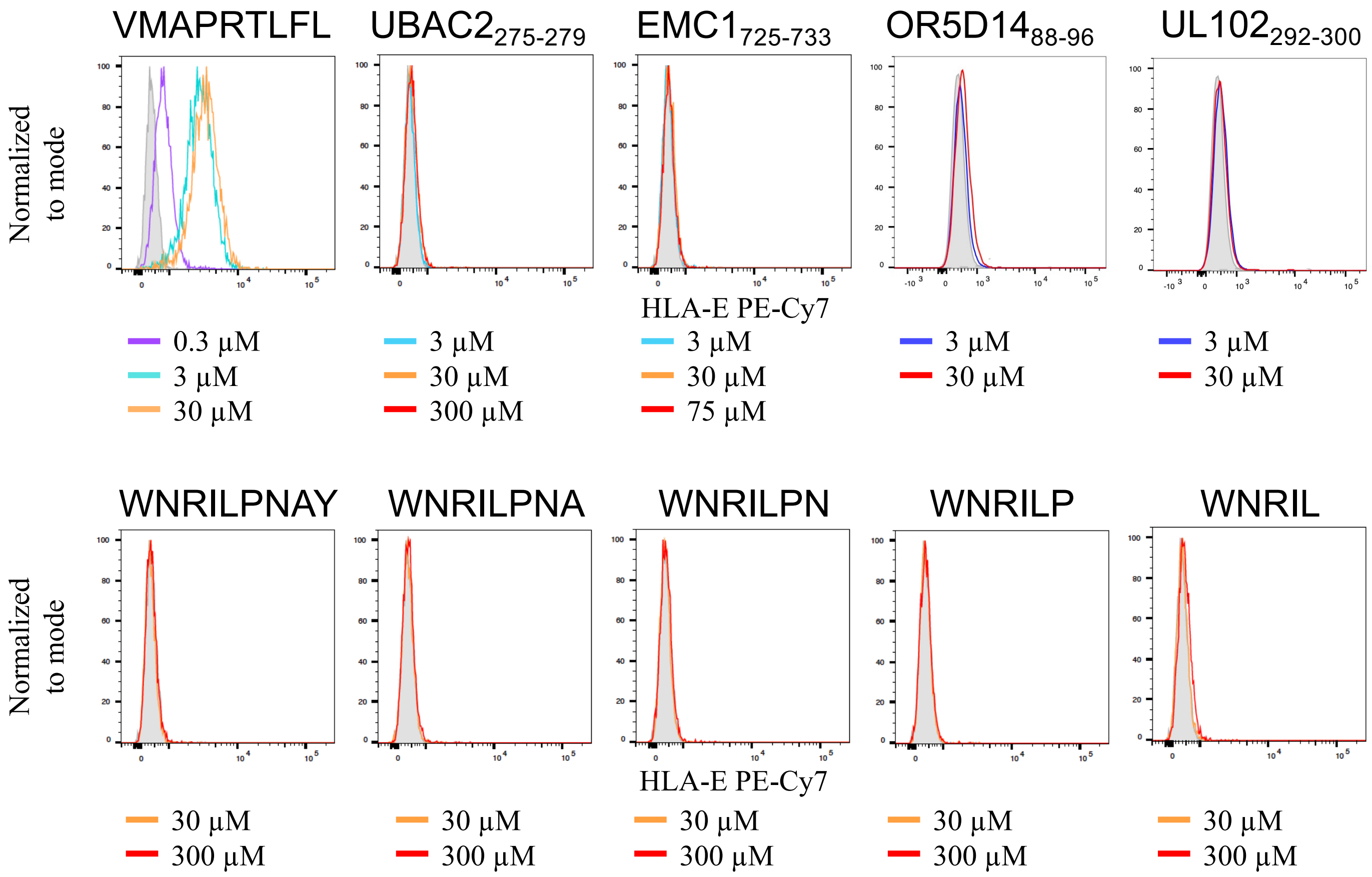

Supplemental Figure 10. Stabilization data for peptides which did not stabilize HLA-E surface expression. Peptides UBAC2$_{275\text{-}279}$, EMC1$_{725\text{-}733}$, OR5D14$_{88\text{-}96}$, and UL102$_{292\text{-}300}$ were assessed, along with length variants of WN peptides. VMAPRTLFL was included as a control. Peptide concentrations tested are indicated, where maximum concentrations assessed vary due to differing peptide solubilities. Grey shaded curve is DMSO control.

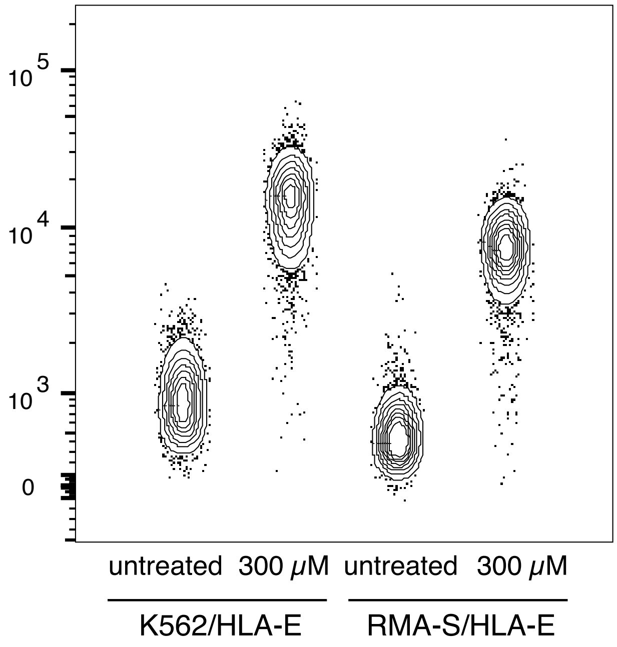

Supplemental Figure 11. HLA-E expression of K562/HLA-E and RMA-S/HLA-E cell lines. HLA-E expression on K562/HLA-E and RMA-S/HLA-E cell lines, determined by anti-HLA-E staining, in the presence of 300 μM VMAPRTLFL peptide or no-peptide control.

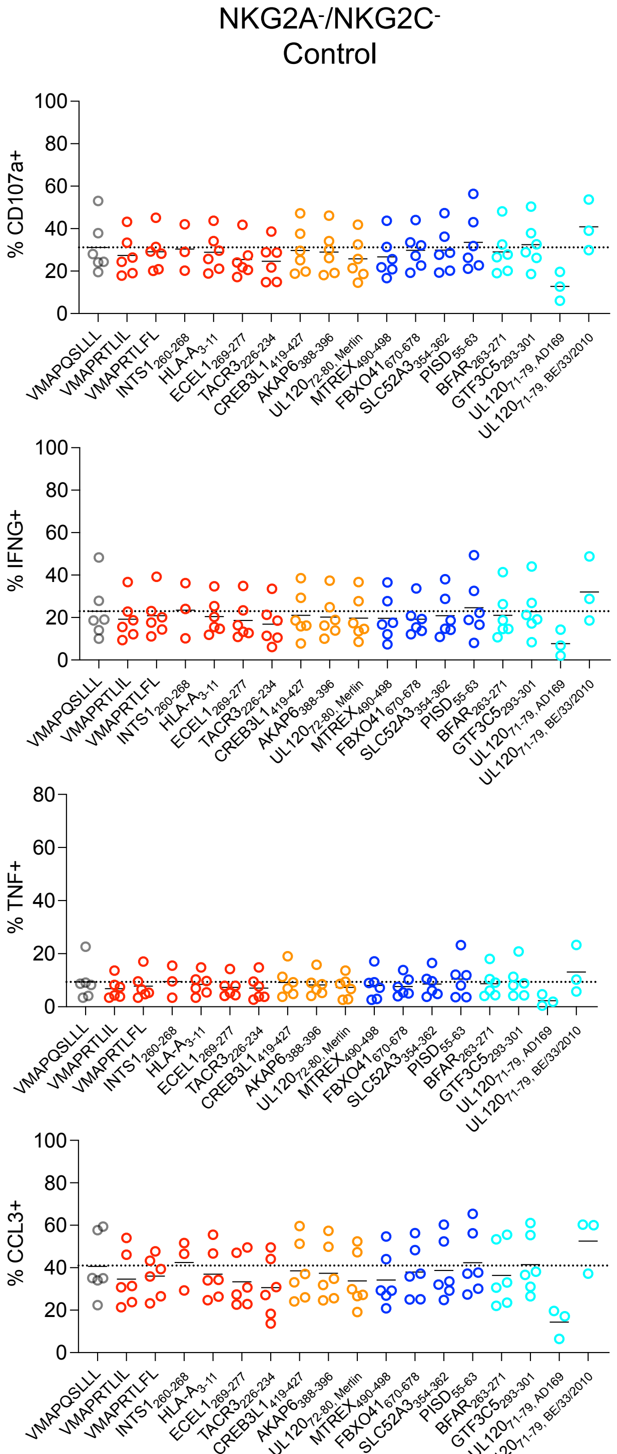

Supplemental Figure 12. NKG2A⁻/NKG2C⁻ NK cell controls. Assessment of effects of peptides on control NKG2A⁻/NKG2C⁻ NK cell activity after co-incubation with peptides and K562/HLA-E cells. Peptides were derived from the human or CMV proteomes or are included as positive (VMAPRTLFL, VMAPRTLIL) or negative controls (VMAPQSLLL). Replicates for individual peptides are from different donors, with solid black lines indicating mean values. Peptides are colored as in Figure 3, highlighting stabilization effects; in order from most to least stabilizing, the colors are: red, orange, blue, and cyan, with the negative control in grey. Representative gating for NKG2A⁻/NKG2C⁻ NK cells is shown in Supplemental Figure 14. Source data are provided as a Source Data file.

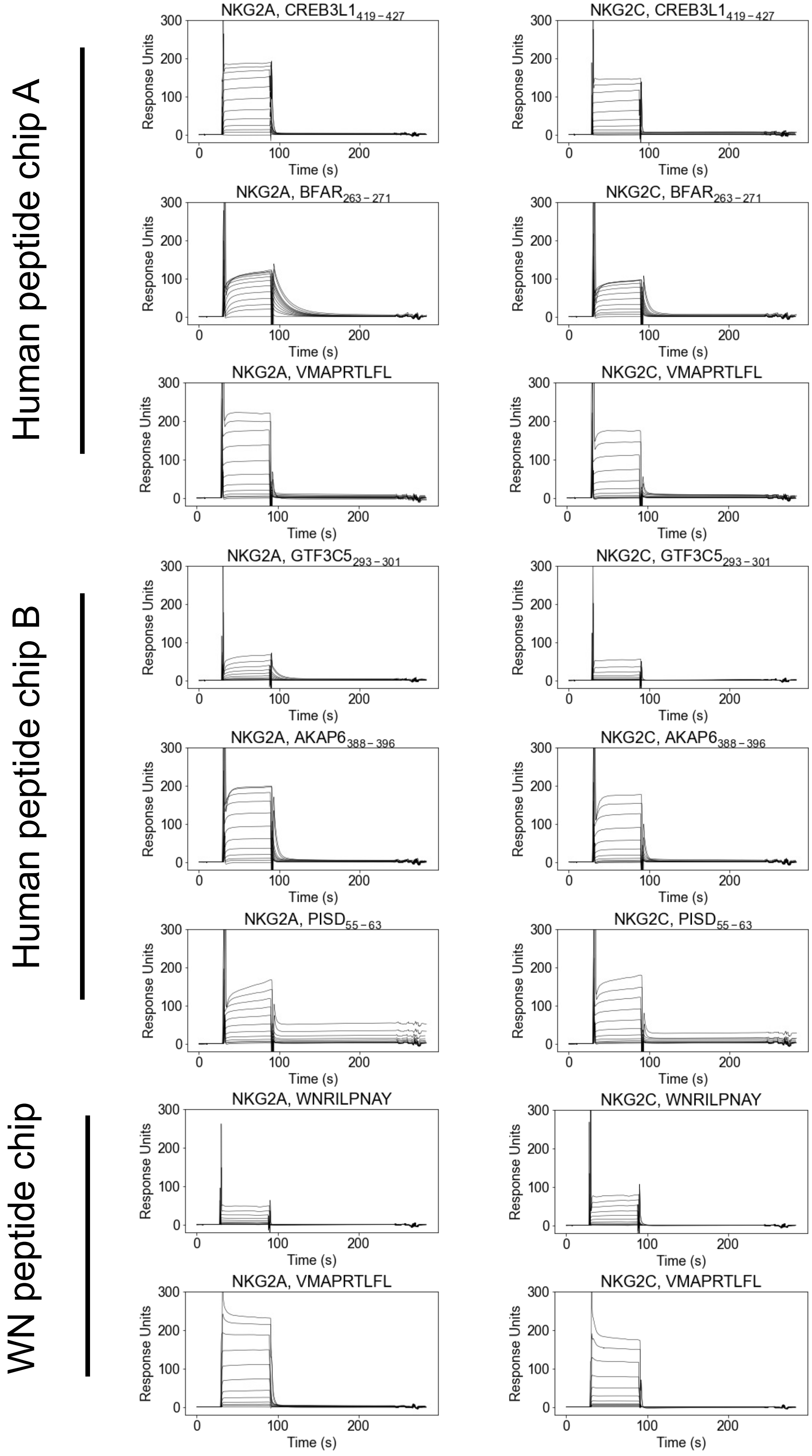

Supplemental Figure 13. SPR sensorgrams. Sensorgrams with peptide, receptor, and chip indicated.

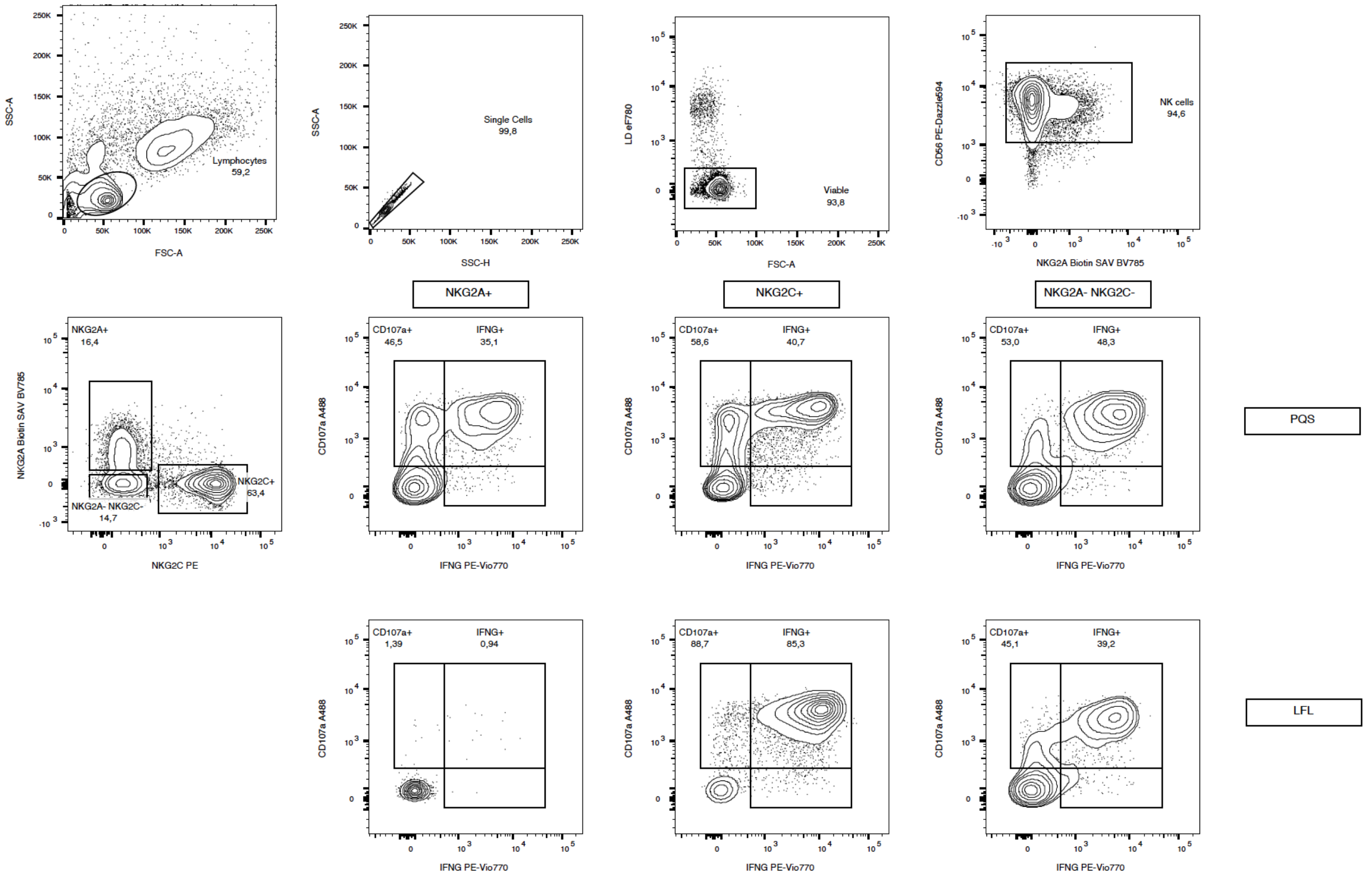

Supplemental Figure 14. Flow cytometry gating. Gating strategy for NK cell stimulation assays.

Antibody	Source	Identifier
PE/Dazzle™ 594 anti-human CD56	Biolegend	AB_2563564 (BioLegend Cat# 318348)
CD3 Monoclonal Antibody (SK7), APC-eFluor 780	Invitrogen	AB_10717514 (ThermoFisher Cat# 47-0036-42)
CD159a (NKG2A) Antibody, anti-human, Biotin, REAfinity	Miltenyi Biotec	AB_2783969 (Miltenyi Biotec Cat# 130-114-090)
CD159c (NKG2C) Antibody, anti-human, PE, REAfinity™	Miltenyi Biotec	AB_2751866 (Miltenyi Biotec Cat# 130-119-814)
Alexa Fluor® 488 anti-human CD107a (LAMP-1) Antibody	Biolegend	AB_1227504 (BioLegend Cat# 328610)
IFN-γ Antibody, anti-human, PE-Vio® 770, REAfinity™	Miltenyi Biotec	AB_2652240 (Miltenyi Biotec Cat# 130-109-235)
Brilliant Violet 605™ anti-human TNF-α Antibody	Biolegend	AB_2563884 (BioLegend Cat# 502936)
CCL3 (MIP-1α) Antibody, anti-human, REAfinity™	Miltenyi Biotec	AB_2651376 (Miltenyi Biotec Cat# 130-103-630)
PE/Cyanine7 anti-human HLA-E Antibody	Biolegend	AB_2565263 (BioLegend Cat# 342608)
PE anti-human HLA-E Antibody	Biolegend	AB_1659250 (BioLegend Cat# 342603)
Alexa Fluor ® 647 HA-Tag Mouse mAb	Cell Signaling Technology	Cell Signaling Technology Cat# 3444S
Alexa Fluor ® 488 HA-Tag Mouse mAb	Cell Signaling Technology	Cell Signaling Technology Cat# 2350S
PE DYKDDDDK Tag (D6W5B) Rabbit mAb	Cell Signaling Technology	Cell Signaling Technology Cat# 98533S
PE anti-Streptavidin Antibody	Biolegend	AB_2571914 (BioLegend Cat# 410503)

Supplemental Table 1. Antibodies used in this work.
